# Assessing the health hazard originated via pesticide chemicals in human through rabbit model in agricultural production system in Bangladesh

**DOI:** 10.1186/s40360-022-00567-5

**Published:** 2022-04-28

**Authors:** Amir Hossan Shaikat, Shahneaz Ali Khan, Mohammed Ashif Imtiaz, Md Ridoan Pasha, Jabin Sultana, Arfanul Saif, Mohammad Rashedul Alam

**Affiliations:** 1 Department of Physiology, Biochemistry and Pharmacology, Chattogram Veterinary and Animal Sciences University, Chattogram, Bangladesh; 2 Faculty of Veterinary Medicine, Chattogram Veterinary and Animal Sciences University, Chattogram, Bangladesh

**Keywords:** Health hazard, Agriculture, Rabbit, Pesticide

## Abstract

**Background:**

The use of chemical pesticides in developing countries like Bangladesh and their impacts on human health and food security is a global concern. Bangladesh is an agricultural dependent country for the growing population demand for food security and food safety. We conduct this study to assess public health threats of commonly utilised pesticides including malathion and nitrobenzene in female rabbit model.

**Methods:**

Thirty New Zealand White healthy rabbit was divided randomly into three groups; and subjected to distilled water as control, malathion@ 5 mg/kg body weight and nitrobenzene@ 5 mg/kg body i.p daily for the next 15 days. Hematology, serum biochemistry and hormonal assay were performed.

**Results:**

Red blood cell (RBC) concentrations (TEC, Hb, PCV%) were reduced in rabbits exposed to malathion and nitrobenzene. The neutrophil and eosinophil percentage were increased in the malathion and nitrobenzene exposed juvenile rabbit group. We found that serum aspartate aminotransferase (AST) and creatinine were increased in the nitrobenzene exposed group in infants, whereas malathion exposure increased serum alanine aminotransferase (ALT). In contrast, the juvenile group exposed to malathion increased the ALT level. There was no change in AST or creatinine levels in juvenile rabbits exposed to malathion or nitrobenzene. Serum estradiol levels were significantly lower in rabbits exposed to malathion and nitrobenzene. Serum testosterone concentration was increased in juvenile rabbits exposed to malathion and nitrobenzene, but progesterone was decreased in malathion exposed juvenile rabbits.

**Conclusion:**

However, this study highlights the importance of rigorous monitoring and testing of agricultural products. In addition, strengthen research and extension in the fields of agro economy, organic farming, local universities and farmer associations.

## Background

Bangladesh rank as the world’s eighth and Asian fifth-most populous country with a land area of 148,456 km^2^, which has an enormous impact on the sustainability of the agricultural production system [1]. However, now this agricultural production system in the country is in the shifting of diversifying from subsistence rice production into higher-value crops like vegetables and fruits [2].

As an agricultural-based country with limited land resources, a massive population to feed, a low and middle-income (LMC) country like Bangladesh is heavily dependent on the utilization of pesticides to increase crop yields. Various research findings revealed the presence of extremely high concentrations of organophosphate and carbamate pesticides in Bangladesh soil and water [3].

In addition, several reports confirmed that about 77% of farmers used pesticides at least once in their crop production cycle [4]. However, several chemicals are extensively used for maximizing plant yield production. These chemicals could be pesticides that protect crops and grains from pests [5], as for high yielding crops or plant energizers or flowering stimulants which increase plant productivity. Though the application of pesticides is targeted to selective toxicity to certain organisms, they could pose serious deleterious effects on non-targeted species, including humans, and might be deposited in the environmental niche. Pesticides from agricultural crop fields wash out into the natural water bodies and adversely affect the quality of water through causing toxicity in many aquatic organisms, including fish as well [6].

Among the pesticides, organophosphates are associated with growth and reproductive abnormalities in animals [7]. Malathion is an organophosphate pesticide that attributes its toxic effect by the inhibition of acetylcholinesterase. However, this pesticide is now being considered an International Agency for Research on Cancer (IARC) probable carcinogen which has been reported to have deleterious effects on humans and animals [8].

Besides that, nitrobenzene is one of the plant energizers and flowering stimulants whose use has been increased in paddy land and lemon gardens in agricultural fields [9]. Unfortunately, nitrobenzene has been recorded as a carcinogen to animals as well as humans [10]. In addition, the contamination of pesticides and other chemicals into water and the eco-system could adversely affect the health of animals as well as humans [11]. Humans generally exposed to this pesticide-based poison via accidental, suicidal or homicidal intoxication [12], and malathion stands at the top in Bangladesh context [13]. But the effects of these chemicals in animals is yet to be understood. Earlier study in Bangladesh and elsewhere has demonstrated the effect of this chemicals in different species that included common carp [14], mice [15], human [16] etc. The Brown Plant Hopper (BHP) outbreak in 1991 in Nilphamari and Rangpur districts of Bangladesh has been attributed to the excessive use of aerial pesticides where cows, calves, goats, fish, honey bees, dragon flies, lady beetles, birds and many other useful insects and animals had been seriously affected [17].

To the best of authors knowledge, no study has been undertaken focusing detail physiological alterations related to hematology, serum chemistry and reproductive hormonal parameters in animal model which could be translated to human health in Bangladesh. For this, here we investigated the physiological changes caused by experimental exposure to malathion and nitrobenzene in rabbits as an animal model. Hence we studied hematology, selective biochemical marker of liver and kidney functions, and some hormonal parameters in experimental malathion and nitrobenzene-exposed rabbits.

## Methods

### Animals

Thirty New Zealand White healthy rabbit (15 was 30–40 days of age (hereafter named as “baby”); 15 was 90–105 days of age (hereafter named as “juvenile”)) was collected from a commercial rabbit rearing farm in Muktagacha, Bangladesh. Rabbits were housed in individual cages with unique identification numbers in their standard housing conditions of 25 °C room temperature and 50% humidity [18]. The rabbit was fed with *Durva* grass (*Cynodon dactylon*) *ad-libitum* and a standard rabbit ration with fresh vegetables and fresh drinking water. Rabbits were given two days of settling time in this new environment.

### Treatment

The baby group of rabbits was divided randomly into three groups (5 in each). After two days of settling time in the new environment, one group was administered with malathion@ 5 mg/kg body weight intraperitoneally (i.p.) daily for the next 15 days. Another group was administered with nitrobenzene@ 5 mg/kg body i.p. daily for the next 15 days. The other group was administered with distilled water i.p. daily for the same period, which served as a control. The juvenile group of rabbits was also randomly divided into three groups, with five in each. The same operational procedures were done for the juvenile group as for the baby group.

### Sample collection, hematology, serum biochemistry, and hormonal assay

Blood was collected from the heart by heart puncture and 2 ml of blood was collected. For hematological studies, 1 ml of blood was kept in a heparinized vacutainer, and 1 ml of blood was kept in a vacutainer without anticoagulant for serum separation. Heparinized blood was run by a hematoanalyzer (Celltac α, Nihon Kohden, Japan) for estimation of total erythrocyte (TEC), total leukocyte count (TLC), hemoglobin (Hb), differential leukocyte count (DLC), erythrocyte sedimentation rate (ESR), packed cell volume (PCV), and other parameters. Serum was separated from coagulated blood (centrifuged at 3000 rpm for 30 min) and analyzed for selective organ function tests (Liver-aspartate aminotransferase (AST), alanine aminotransferase (ALT), and Kidney-Creatinine) using a biochemical analyzer (Humalyzer-3000) with the commercially available kits. Reproductive hormone parameters such as FSH (NovaTec Immundiagnostica GmbH, Germany), LH (Atlas Medical, Germany), Estrogen (NovaTec Immundiagnostica GmbH, Germany), Progesterone (NovaTec Immundiagnostica GmbH, Germany), Testosterone (BIOS Microwell diagnostic system, Netherlands) have been checked by a specific ELISA kit. The procedures were performed according to the manufacturer’s instructions of the respective ELISA kit. A four-parametric logistic curve fit was performed to analyze hormone concentration.

### Data analysis

Data was stored in Microsoft Excel and ANOVA was deployed to compare the effects of malathion and nitrobenzene with the control group. The findings were expressed in mean ± standard error of the mean (SEM). The level of significance was set at p˂0.05.

## Results

### Hematology alteration

In the baby group, TEC was significantly decreased in malathion and nitrobenzene exposed rabbits and Hb, and PCV was decreased in malathion exposed rabbits. Besides, TLC and lymphocytes were increased in the malathion exposed group, but it decreased the neutrophil percentage (Table 1). In the juvenile group, the same effect of malathion was observed in the blood parameters, as was observed in the baby group. In addition, both malathion and nitrobenzene increased neutrophil and eosinophil percentages (Table 1).

**Table 1:** Hematological estimation of rabbit after chemical stimulant and pesticide exposure

Parameter	Control	Malathion	Nitrobenzene
Baby group			
TEC (million/cumm)	5.62 ± 0.17^a^	4.49 ± 0.11^b^	4.87 ± 0.18^b^
TLC (thousand/cumm)	3.6 ± 0.18^b^	4.1 ± 0.13^a^	3.4 ± 0.02^b^
Hb (gm/dl)	8.72 ± 0.03^a^	8.12 ± 0.03^b^	8.44 ± 0.25^ab^
PCV (%)	40 ± 0.54^a^	34.84 ± 2.09^b^	38.06 ± 0.7 ^ab^
ESR (mm in 1^st^ hour)	0.1 ± 0.1	0.1 ± 0.1	0
Neutrophil (%)	27.2 ± 1.49^a^	20.4 ± 1.83^b^	24.2 ± 1.74^ab^
Eosinophil (%)	6 ± 0.83	6.6 ± 1.2	6 ± 0.54
Basophil (%)	1.4 ± 0.5	0.4 ± 0.24	0.4 ± 0.24
Monocyte (%)	8.8 ± 1.06	7.2 ± 2.03	6 ± 1.97
Lymphocyte (%)	56.6 ± 1.07^b^	65.6 ± 1.32^a^	63.4 ± 0.6^a^
Juvenile group			
TEC (million/cumm)	5.57 ± 0.14^a^	4.6 ± 0.16^b^	5.21 ± 0.18^ab^
TLC (thousand/cumm)	3.6 ± 0.07 ^b^	4.34 ± 0.25 ^a^	3.84 ± 0.21 ^ab^
Hb (gm/dl)	10.9 ± 0.32^a^	8.48 ± 0.13^b^	10.1 ± 0.43^a^
PCV (%)	34.24 ± 0.54^a^	30.38 ± 0.58^b^	33.9 ± 1.27^a^
ESR (mm in 1^st^ hour)	0	0	0
Neutrophil (%)	24.4 ± 0.4^b^	36.6 ± 0.67^a^	37.6 ± 3.61^a^
Eosinophil (%)	6.6 ± 0.4^b^	24.6 ± 1.5^a^	20.4 ± 2.76^a^
Basophil (%)	0.2 ± 0.2	0.6 ± 0.2	0.2 ± 0.2
Monocyte (%)	1 ± 0.63^b^	4 ± 1.58^b^	8.8 ± 0.58^a^
Lymphocyte (%)	67.4 ± 0.92^a^	34.2 ± 2.83^b^	33.2 ± 5.59^b^

Different superscripts (^a,b,c^) in same row means significantly different (*p*˂0.05)

### Serum biochemical alteration

We concentrated on some vital organ functional changes caused by malathion and nitrobenzene exposure. We found serum AST and creatinine were increased in the nitrobenzene exposed group in the baby group, whereas malathion increased serum ALT. In contrast, the juvenile group exposed to malathion increased ALT levels. There was no change in AST or creatinine levels in rabbits exposed to malathion or nitrobenzene (Table 2).

**Table 2:** Serum biochemical concentration of rabbit after chemical stimulant and pesticide exposure

Parameter	Control	Malathion	Nitrobenzene
Baby group			
AST (U/L)	113.36 ± 8.35^ab^	86.84 ± 16.26^b^	155.84 ± 10.41^a^
ALT (U/L)	34.68 ± 1.05^b^	53.9 ± 1.43^a^	37.48 ± 0.98^b^
Creatinine (mg/dL)	0.54 ± 0.02^b^	0.46 ± 0.02^b^	0.86 ± 0.02^a^
Juvenile group			
AST (U/L)	132.04 ± 12.5	157.48 ± 32.14	145.52 ± 20.92
ALT (U/L)	79.96 ± 1.98^b^	91.64 ± 2.6^a^	79.66 ± 2.88^b^
Creatinine (mg/dL)	0.76 ± 0.07	0.71 ± 0.04	0.68 ± 0.03

Different superscripts (^a,b,c^) in same row means significantly different (*p*˂0.05)

### Serum hormonal alteration

Serum estradiol levels were significantly reduced in both malathion and nitrobenzene-exposed baby and juvenile rabbits. Serum testosterone levels were increased in juvenile rabbits exposed to malathion and nitrobenzene, but progesterone concentrations were decreased in malathion-exposed juvenile rabbits (Table 3).

**Table 3:** Hormonal alteration of rabbit after chemical stimulant and pesticide exposure

Parameter	Control	Malathion	Nitrobenzene
Baby group			
FSH (mU/mL)	0.11 ± 0.01	0.11 ± 0.01	0.11 ± 0.01
LH (mIU/mL)	0.01 ± 0.001	0.01 ± 0.001	0.01 ± 0.002
Estradiol (pg/mL)	22.48 ± 0.45^a^	16.53 ± 0.35^b^	17.31 ± 0.53^b^
Progesterone (ng/mL)	8.47 ± 0.31	8.10 ± 0.31	8.10 ± 0.32
Testosterone (ng/mL)	0.03 ± 0.01	0.04 ± 0.01	0.04 ± 0.01
Juvenile group			
FSH (mIU/mL)	0.20 ± 0.01	0.20 ± 0.01	0.19 ± 0.01
LH (mIU/mL)	0.02 ± 0.001	0.02 ± 0.002	0.03 ± 0.001
Estradiol (pg/mL)	37.34 ± 0.40^a^	31.87 ± 0.52^b^	31.67 ± 0.75^b^
Progesterone (ng/mL)	10.78 ± 0.21^a^	6.99 ± 0.21^c^	8.40 ± 0.32^b^
Testosterone (ng/mL)	0.04 ± 0.01^b^	0.18 ± 0.02^a^	0.17 ± 0.01^a^

Different superscripts (^a,b,c^) in same row means significantly different (*p*˂0.05)

## Discussion

### Slight deviation of hematology / consistent hematology values in neonatal and juvenile rabbit

Hematological parameters such as TEC, Hb, and PCV were significantly decreased in malathion exposed baby and juvenile rabbits might be because of failing of hematopoietic system in rabbit exposed to malathion compared to control group and nitrobenzene exposed group. As toxicants exposure exerts an adverse effect on the hematopoietic organs which in turn could alters blood parameters [14]. A significant decrease in Hb concentration to malathion may be due to less oxygen supply to different tissues, resulting in a sluggish metabolic rate and low energy output [19]. This decline in Hb and RBC values could happen due to the breakdown of iron synthesizing machinery [20].

PCV readings are important in determining the effect of pesticides and in determining the oxygen-carrying capacity of the blood. Previous studies [14, 19–24] found a similar decrease in TEC and Hb in malathion and nitrobenzene-exposed animal models.

The host immunological response and defense mechanisms are usually maintained through the activation of WBC. Therefore, the fluctuations of the WBC number in the blood vascular system indicate an abnormality from an immunological perspective. In our study, WBC/TLC significantly increased with increasing the toxicity of malathion as an adaptive measure for the tissue under the toxicant exposure. This exposure exerts an adverse effect on the hematopoietic organs, which in turn alters blood parameters. Malathion exposure remarkably increases the blood’s circulating white blood cells [19, 25]. Increased WBC count resulted in leucocytosis, which is thought to be adaptive for the tissue under chemical stress. This also helps in the removal of cellular debris from necrosed tissue at a faster rate [26]. In the presence of foreign substances or under pathological conditions, leucocytosis in fish may be the consequence of direct stimulation of immunological defense [26]. The increase in leucocyte count can be correlated with an increase in antibody production which helps in survival and recovery of the fish exposed to malathion [27].

In our study, the neutrophil and eosinophil concentrations were significantly increased in both malathion and nitrobenzene exposed animals. These cells play a key role in combating a great variety of weapons and diverse chemicals as chemotactic triggers that are directed against different kinds of pathogens. The granules secreted from eosinophils and neutrophils into the extracellular space upon activation consist of a variety of important mediators that are crucial for the function of granulocytes within innate immunity [28].

### Significant alterations of ALT and AST two key liver and kidney functional enzymes in both neonatal and juvenile rabbit

Malathion may change the functions of vital organs like the liver and kidney, disrupting the homeostatic phenomenon of the body. The altered serum AST, ALT, and creatinine in our study could be due to increased malondialdehyde MDA formation. MDA is formed in liver cells through a lipid peroxidation process that enhances mutagenicity and inactivates enzymes by altering lysine residues. In rats, following a deltamethrin oral dose elicits increased MDA formation and decreased activities of CAT, SOD, and GSH levels [29]. This process could develop through free radical [O2-] formation as deltamethrin undergoes metabolism in the liver via hydrolytic ester cleavage and oxidative pathways by oxidative damage [30]. The metabolism of various drugs, chemicals, and pesticides is primarily responsible for the onset of biochemical and histopathological changes in the liver involving cytoplasminolysis, nuclear pyknosis, and necrosis of hepatocytes [31]. However, Nitrobenzene carcinogenicity follows similar metabolic pathways to form a number of phenolic compounds that play a more potent role in Nitrobenzene carcinogenicity. The increased concentrations of reactive oxygen species (ROS) cause obvious tissue damage and oxidation of lipids, proteins, and DNA. The increase in AST concentration in plasma is clearly an indication of cellular damage. The damaged hepatic cell membrane through necrosis leads to the release of their enzymes into the circulation [32]. Previous studies also demonstrated malathion and nitrobenzene induce liver and kidney dysfunction by altering the AST, ALT, and creatinine levels in various animal models [10, 33–37].

### Major impact on steroidal hormone alterations in both age groups of rabbit

The major effects of pesticides in developing and developed countries represent a great benefit for human health through controlling agricultural pests and disease vectors, human and livestock disease vectors, and nuisance organisms. Moreover, they ensure increased food production for the growing population’s demand. However, many first-generation pesticides have been found to be harmful to the environment. Some of them can persist in soils and aquatic sediments and have a residual impact on top predators and human health via bio-concentrates in the tissues of invertebrates and vertebrates. In our study, the estradiol concentrations were reduced significantly in malathion and nitrobenzene-treated animals. Interestingly, serum testosterone was increased in concentrations in juvenile rabbits, though all animals fell into the female group. Malathion and γ-BHC had toxic effects on the metabolism of sex steroids and disturbed steroid regulation, resulting in hormonal imbalance [38]. Both pesticides caused a greater reduction in sex hormone levels at higher concentrations. Follicles are the principal functional units of the mammalian ovary. Follicle stimulating hormone (FSH) and luteinizing hormone (LH) are the important controllers of follicle development. FSH and LH are produced by the pituitary gland and the ovarian steroid by granulosa cells of the ovarian tissue. In previous studies, the number of healthy follicles was significantly decreased with a concomitant increase in atretic follicles and corpus luteum areas being developed in greater dimension by malathion and nitrobenzene exposure [39, 40]. In our study, we did not observe these types of tissue-level changes, but the estradiol concentration was greatly reduced. In another study, with malathion exposure in rats, the FSH concentration was significantly increased but the estrogen level dramatically decreased [41]. Previous studies on malathion and nitrobenzene also demonstrated similar findings in female chickens and fish where estrogen and progesterone levels were severely decreased but the testosterone concentrations were increased as in our present study [42, 43]. Reduced estradiol levels in malathion and nitrobenzene-exposed rabbits in our study could result in fewer healthy ovarian follicles, granulosa cell apoptosis, and ovarian anomaly, as seen in other animals [39, 40].

In summary, pesticide exposure has a great impact on the sex hormone metabolism process and subsequently deteriorates its regulation through fluctuations in its concentrations as well.

### Residual impact of pesticides on public health concern

Currently, the agricultural production system enhances the utilization of several pesticides that have been associated with health and environmental issues, and on the other hand, the agricultural use of certain pesticides has been abandoned. People have been exposed to pesticide chemicals through contact with their skin, ingestion, or inhalation [44]. The type of pesticide, the duration and route of exposure, and the individual health status are determining risk factors in the possible health outcome in a particular community. Pesticides or chemicals might be metabolized into more carcinogenic compounds within the human or animal body or may be excreted via different routes, stored, or bioaccumulated into body fat [45]. US epidemiologists observed an unusual rise in Non-Hodgkin’s Lymphoma in areas of high pesticide use [46]. The numerous health effects that have been associated with pesticides include dermatological, gastrointestinal, neurological, carcinogenic, respiratory, reproductive, and endocrine effects [47]. The major impact of pesticides could be its residual effect through accumulating in everyday foods and beverages, including cooked meals, water, meats, milk, eggs of animal origin, vegetables, and animal feed. Furthermore, it’s an alarming issue that washing and peeling could not be able to remove the residual chemicals from human food sources [48]. Exposure to organophosphorus pesticides could be a risk factor for congenital anomalies and other adverse birth outcomes [49, 50]. In the context of safety levels, the concentrations might not exceed the legislatively determined safe levels, though no research has been implemented in Bangladesh yet. However, these "safe limits" may underestimate the practical aspect of the pesticides’ residual impact on human health. Although the agricultural soil is the primary recipient of agrochemicals, water bodies that are adjacent to agricultural areas could be the ultimate recipient for agrochemicals residues. Therefore, agrochemicals residues are common in surface water system, especially in irrigation drains, which ultimately pollute the water bodies and can harm aquatic environment [51], animals and human exposed to it. A study of pesticide residues in some selected ponds of Bangladesh showed the residue level of malathion was 0.0241 to 0.463 ppm, carbofuran was 0.0302 to 0.0629 ppm and cypermethrin (pyrethroid) was 0.0141 to 0.09 ppm [52]. In addition, the identification of pesticides in human breast milk highlights the importance of public health concerns originating from residual pesticides. Our study only include female rabbits which also explored indirectly the health hazard in human women as they are more susceptible to various cancer and vulnerable to environmental pollutants as well as pesticides [53, 54].

## Conclusion

Modern agricultural practices in developed and developing countries, including Bangladesh, utilize extensive chemicals for increased population demand and a variety of production processes. These chemicals could cause deleterious effects on living individuals, as proved in this experiment, causing defective liver and kidney function and hormone production. Current agriculture practices have to implement environmentally friendlier practices that pose fewer public health risks for the millennium development goals (MDGs) for a developing country like Bangladesh.

## Data Availability

The datasets used and/or analysed during the current study available from the corresponding author on reasonable request.
